# Shaping Physical Activity through Facilitating Student Agency in Secondary Schools in the Netherlands

**DOI:** 10.3390/ijerph19159028

**Published:** 2022-07-25

**Authors:** Gwendolijn M. M. Boonekamp, John A. J. Dierx, Erik Jansen

**Affiliations:** 1 School of Sport and Exercise, HAN University of Applied Sciences, P.O. Box 6960, 6503 GL Nijmegen, The Netherlands; 2 Research Group Living in Motion, AVANS University of Applied Science, P.O. Box 90.116, 4800 RA Breda, The Netherlands; jaj.dierx@avans.nl; 3 Research Centre for Social Support and Community Care, HAN University of Applied Sciences, P.O. Box 6960, 6503 GL Nijmegen, The Netherlands; erik.jansen@han.nl

**Keywords:** agency, adolescent students, physical activity, social practice theory, whole-school approach, salutogenesis

## Abstract

Schools can enable students’ capability for lifelong physical activity (PA) by providing a physical, social and pedagogical context fostering students’ agency. This entails allowing them to develop their autonomy and ability to shape PAs according to what they value. This paper explores *whether*, and, if so, *how,* school practices provide the conditions for developing and employing PA-related student agency. We followed a multiple case study design, partnering with six secondary schools in the Netherlands. We based our qualitative content analysis on the schools’ policy documents and transcripts of interviews and focus groups with school management, teaching staff and supporting sports professionals. First, we analysed the space provided for PA-related student agency using the whole-of-school framework. Next, we used a social practice theory perspective to understand how each school practice allows for student agency. Results suggest that schools offer sufficient and varied PA opportunities but have not embedded deliberation with students on their meaningfulness and transfer to other contexts in their practices. We conclude that for schools to promote lifelong PA for students, there is a need for a pedagogical approach involving students’ perspectives, participation and reflections, enabling them to transfer PAs to other (future) life settings.

## 1. Introduction

Schools can play a crucial role in preparing students for lifelong physical activity (PA). For instance, whole-school multiple component approaches (WSA), as well as the Health Promoting Schools framework of the World Health Organization [1,2], have been successful in increasing student PA in schools, reaching the recommended 60 min of required PA [3,4,5]. In these approaches, school environments provide PA-enticing infrastructures, besides the formal curriculum, to support PA throughout the day as well as supportive policies and an engaged school community [6,7]. They also include facilitating PA in and around school with transport, physical education classes, recess, classroom activities and pre- and after-school programs. Nevertheless, worldwide, 81% of adolescents (aged 11–17 years) fail to meet the recommended guidelines, including in the Netherlands (80%) [8]. Therefore, alternative or complementary school strategies are needed, especially when promoting lifelong PA for young people.

Inspired by Morin’s [9] views on education, such alternative or complementary strategies may include: (i) enabling students to try and discover enjoyable PA experiences that are meaningful to them, and (ii) supporting them to contextualise their experiences, enabling them to translate and integrate PA into their daily lives and contexts outside of the school.

These strategies resonate with salutogenic theory [10,11]. Its key concept, sense of coherence (SoC), clarifies the relation between students’ appreciation of PA, their understanding (comprehensibility) and use of their PA options (manageability) and whether they are related to the aspects and goals they value (meaningfulness) [12,13]. Furthermore, a salutogenic approach in organising PA in schools entails: (i) strengthening resources for all students and not just those deemed at risk; (ii) developing students’ salutary factors in terms of their dispositions and skills, and their ability to transfer their practices to other contexts; and (iii) focusing on the whole person of the student, which includes the relevance of their lives, experiences and contexts, and life histories [14,15].

In turn, the SoC resonates with findings based on self-determination theory (SDT): that behaviour is better internalised when students are provided with a meaningful rationale, choice options and perspective-taking by their teachers [16,17,18]. These studies concur with the value of inspiring students and providing them with choice options and support for motivation toward similar activities outside school [19]. In this way, students develop a so-called *agency* for lifelong PA. The innovative notion underpinning the current study is the beneficial value of school environments focusing on fostering this students’ agency.

### 1.1. Students’ PA-Related Agency

Students can become change agents in and of their contexts, which fits Agenda 2030 [20]. For students to develop their capacity to construct their meaning-making regarding PA and become vital, resilient citizens who act in an ethically responsible way, their education should address academic, civic and critical thinking competencies, increasing their health literacy [21,22,23]. However, students’ contexts tend to impact their PA critically [8,24]. Therefore, encouraging students to reflect upon their PA experiences in and outside school can advance their understanding, meaning-making and transferring and transforming abilities to different contexts such as their neighbourhoods, homes or sports clubs [9,25,26]. It requires acknowledging students as active participants in various complex social systems that are interlinked, and of which the elements mutually influence each other [26,27].

In a previous paper [28], we argued for schools to focus on developing students’ PA-related agency. Such focus enables students to discover what PAs fit with their perspectives and aspirations and facilitate the transfer to their daily lives. The notion of ‘agency’ is derived from the capability approach in which it is considered a driving force in a person’s pursuit of a flourishing life [29,30]. We combined salutogenic theory with Freire’s and Morin’s views on the role of education, and with Claassen’s capability theory of social justice to conceptualise student PA-related agency. ‘Students should want to (autonomy) and be able to (capacity) pursue their own life goals, and feel encouraged by their socio-cultural and physical context to include PA in their own life goals (experience freedom) to engage in lifelong PA’ [28]. Four essential elements follow from this conceptualisation: (i) a student’s awareness of contextual resources and options to achieve goals; (ii) contextual choice options to freely use and choose from; (iii) abilities to set personally valued goals and to choose adequate means to pursue these goals; and (iv) capacities to achieve the selected personally valued goals. Thus, we view students’ agency as a dynamic interplay of a student’s characteristics and the contextual options and resources in school. Schools facilitating agency provide an inclusive context where all students can sufficiently develop such agency by developing autonomy and exercising freedoms [28].

According to Claassen [29], agency is an attribute of individuals which is simultaneously socially embedded. He distinguishes ‘participatory’ and ‘navigational’ agency, referring to mobility within and between social practices, respectively. Navigational agency is particularly relevant for schools aiming to enhance students’ agency for physically active lifestyles across the lifespan in different contexts. We interpreted participatory and navigational agency as follows:

- Offering PA choice options and opportunities to shape PAs can contribute to students’ *freedom* to participate, or not, in existing practices;
- Reflection upon PA experiences contributes to *autonomy* in weighing options and possibilities of participating in PA;
- Enabling the transfer of PAs to other contexts contributes to students’ navigational agency by facilitating the adaptation of practices in these different contexts.

### 1.2. ‘Space’ for Students’ Agency in Schools as Social Practice

School contexts can be considered as social practices where PA-related learning occurs. In these practices, clusters of complex interactions occur between elements such as the school’s policy, physical characteristics and infrastructure of the premises, and social, cultural, geographical, historical, pedagogical and managerial elements [1]. Morin emphasises the need to take account of the connections between elements, acknowledging the context and how they influence each other [26]. Elizabeth Shove’s social practice theory [31] is well-placed to study such complexity integrally [32]. Her theory simplifies social practices into a distinction of three categories of elements [31]: *meaning* (i.e., ideas, aspirations and embodied understandings of the social significance of the practice and past experiences of participation in a social practice), *materials* (i.e., objects, consumer goods and infrastructures) and *competencies* (i.e., skills, practical know-how and techniques, including understandings of the situation). In the context of our study, we operationalised these elements as follows:

- Meaning: a school’s vision, mission, and pedagogical approach to their task in general, and allowing for the development of students’ agency in general and PA in particular;
- Materials: the access to infrastructural facilities such as playgrounds, the natural environment and the community around the school, allowing for PAs, including roads and cycling infrastructure, as well as choice options provided in PAs in lessons, during breaks or before and after school;
- Competencies: the management style and priorities, skills and practical know-how of teachers, staff, municipal sports professionals and other stakeholders in the school practice and community to provide conditions for developing PA-related student agency.

The data used in this study are part of a larger, longitudinal, experimental project in 22 secondary schools in the Netherlands between 2014–2017 by two universities of applied sciences involving their students as co-researchers. The larger project aimed to provide input for developing PA-enhancing strategies in schools by aligning them with the perspectives and assets of students. In addition to insight into students’ perspectives on their PA-related agency [12], we were interested in whether schools provide the conditions for developing PA-related student agency. The present study aims to inform PA-enhancing strategies in schools by gaining insight into *whether* and *how* schools provide the conditions for the development and exercise of PA-related student agency. Therefore, our research question is: How can our propositions of participatory and navigational agency be recognised in the elements of the Whole-School Approach in six of the initially 22 participating schools and how do they relate to the three elements of social practice?

## 2. Materials and Methods

### 2.1. Study Design and Contextualisation

We followed a multiple case study design, considering each school as a distinct social practice. Most schools had expressed a prior interest in enhancing healthy lifestyles with students and had often already participated in the Dutch Healthy School project. We designed a study based on a qualitative analysis of school policy documents, interviews and focus groups with school management, teaching staff and supporting sports professionals. Underlying our method is a constructivist ontology and subjectivist epistemology [33]. These assume that people actively construct and then act upon social realities they assign to events, actions, processes, ideologies, and conditions. The notion of multiple realities [33] was key to qualitative data collection and analysis. Strategic policy documents, often covering school alliances, are implemented by school managers and operationalised by teaching staff. We aimed to explore if and how PA-related agency could be recognised in these different realities.

### 2.2. Participants and Recruitment

Six secondary schools from the previously mentioned project were included. All schools except one are geographically located in the Arnhem–Nijmegen area in the Netherlands. The study sample consisted of school managers, chairs of physical education (PE) teachers’ teams, teaching staff and municipal sports professionals (SP).

### 2.3. Data Collection

Data were collected between April and November 2017 and concerned three data sources: school policy documents, semi-structured interviews and focus groups.

A content analysis was conducted of the schools’ strategic *policy documents*, plans, and yearly reports from 2014 to 2020. All schools were part of school alliances responsible for the strategic plans. Individual schools could further interpret, operationalise and prioritise these plans. Most policy documents were publicly available as downloadable pdf-files on the school’s website. Where absent, we contacted school administrators to obtain policy documents via e-mail.

Six individual and one two-person *semi-structured interviews* were conducted as part of the evaluation of the larger project. Before each interview and focus group, we informed participants verbally that we would analyse their views in the context of their school and their colleagues’ views. Participants provided spoken and audio-recorded consent before commencing. Interviews aimed to explore the schools’ policies and plans regarding health, sports and exercise and students’ participation. The interview was based on a questionnaire from the Dutch Healthy School Project [34] and extended to include two questions derived from the rapid assessment tool from the Schools for Health in Europe Network [35]. These combined tools resulted in an interview guide (in Dutch) consisting of the following topics: policies concerning the needs and wishes of the school community regarding active lifestyle, PA and sports; monitoring health issues in students; construction of PE lessons, physical environment regarding PA; and collaboration with PA-related stakeholders in the community. The interviews were audio-recorded and transcribed for further analysis.

In five schools, we conducted *focus groups* with school staff using the narrative timeline technique [36,37]. The sixth school could not organise a focus group due to organisational circumstances. Participants of the focus groups were selected and invited by the school project leader. These sessions took place between June and November 2017, following the interview at that particular school. Each focus group was also conducted on-site. Once we clarified the purpose and procedure of the focus group, we depicted a timeline on a flip chart with three rows. The top row was to reflect on events of positive energy in the process, the middle on events that drained energy, and the bottom row on breakthrough events (particular insights, seized opportunities). Participants could use a marker and sticky notes in three colours: green for energising or inspiring events or occasions, red for energy-draining events, and yellow for experiences of insight or breakthrough. They described these occasions in brief statements that mattered most to them individually. Next, participants placed their sticky notes on the timeline. The researcher clustered statements and facilitated a plenary dialogue, contextualising them. This contributed to mutual understanding of how participants had experienced the process individually, marking milestones in the process (e.g., critical incidents or meetings). Finally, conclusions and action points were noted. Each focus group lasted about 90 min and was audio-recorded and transcribed for further analysis.

### 2.4. Data Analysis

Digital policy documents and transcripts of the interviews and focus groups of the six schools were analysed with Atlas.ti (Version 8, ATLAS.ti Scientific Software Development GmbH, Berlin, Germany). For reasons of privacy while still allowing for identification within our study, the schools were numbered (School 1, School 2, etc.), and the respective policy documents, interviews and focus groups were numbered consistently (pd1, pd2, etc.; i1, i2, etc.; and fg1, fg2, etc.).

First, we developed a framework for analysis, factorially combining the components of the WSA with the two contextual aspects of PA-related student agency. Concerning WSA, we distinguished between *when* (before, during, and after school) and *where* (within which infrastructure) PA takes place and the role staff and the wider school community play in facilitating PA. For agency, we distinguished between the *autonomy* of students to set their own goals and their *freedom* to choose and organise their PAs. Applying this framework led to several labels to code fragments from the three data sources. Quotes across the schools were coded using latent coding, which meant we looked for the message behind the text to group labels together. The results provided us with the first level of analysis: we attributed quotes to the different cells where the whole-school components ‘meet’ the contextual dimensions of agency (Table 1).

Next, to analyse each school as a social practice allowing for PA-related agency, we coded findings from the first analysis following our operationalisation of social practice as defined by Shove [31]: meaning, competencies and materials (Table 2).

## 3. Results

### 3.1. Whole School Approach and Agency Components across Schools

All six school contexts provided their students with opportunities to develop their (PA-related) agency in terms of *autonomy*. We identified a wide variety of aspects and possibilities in the different elements of the WSA in their policy papers and the reflections of school managers, teachers and SPs (see Table 1). Our findings illustrate that schools struggle with the *freedom* aspect of agency: how to enable students to develop this freedom and foster their uptake of agency. Below, we describe for each of the components of the WSA, some noteworthy findings related to both aspects of agency across the six schools.

Concerning PA-related agency in *physical education lessons and before, during and after school*, we found that teachers assume or experience that students are not (yet) capable of formulating their wishes concerning PA. They struggle with balancing what they need to teach their students in terms of motor skills versus expanding students’ horizons beyond what is familiar to them, as illustrated by the following examples. A school manager mentioned that it is the school’s ‘SP who engages students in dialogue who are not yet involved in organised PAs outside the school, trying to support them with this’ (i5). Furthermore, teachers seem to face various dilemmas when considering giving choice options to their students. One group of teachers said: ‘We announce a wide variety of PAs through Instagram. An adult generally designs them and checks the ideas with students. Students generate few ideas themselves. You have to inspire and challenge them’ (fg2). Another teacher’s pedagogical reflection was: ‘I do not know if we should give students only choice options. Sometimes they have to, I think. Maybe that is an old-fashioned thought, but sometimes you have to make students do things to experience things outside their comfort zone. I mean, they have to do something they are not good at or do not like a priori, instead of them always choosing the same things’ (fg5). Regarding the freedom to choose and organise PA, we found that aspects of decision making, reflection and transfer are recognisable but not necessarily purposely addressed or interconnected. Some schools provide choice options and possibilities for students to develop and sometimes teach their own PAs. As a PE teacher mentioned: ‘Our final course students teach PE in groups of 3 students at the local primary school. PE teachers design the lesson, and students look up ways to operationalise the aims of the lesson’ (i3). Another element mentioned was creating student teams based on competence levels: ‘As a school, we participate in many local and national tournaments and create student teams across school levels which contribute to building citizenship’(i5). It is noteworthy that these opportunities are often focused on students intrinsically motivated for PA and are ‘supply-driven (i1)’.

The schools’ *infrastructure* is an aspect that can allow for, or inhibit, PA-related agency. In most schools, the amenities are not freely accessible for students due to policy and safety regulations, other users, or lack of supervision (freedom). Nevertheless, most schools consciously create choice options that allow PA through their premises (autonomy). Examples of structural ways to achieve this included inviting students ‘to provide input for the new school playground. The school organised a competition and invited the three winners to co-design with the school director and architect’ (i4). Another school applied an unused room to create ‘an e-sports room with static bicycles and a dance floor attracting die-hard gamers’ (i4a). Another school invests in a yearly ‘obstacle run and laser game which entails using the whole school building differently’ (i2). As per surrounding infrastructure, schools negotiate with the municipality for safer roads, allowing for active mobility: ‘The municipality made the school’s roads safer with speed bumps’ (i5). One school actively involved its students in this process: ‘students adopt a roundabout or traffic situation, prepare plans for improvement and safety, and present them to the municipality’ (i2).

We found a variety of illustrations concerning the *staff’s involvement* in developing students’ PA-related agency. Whether or not being explicitly part of policy plans, most of the time, it depends on the PE teacher’s approach or the availability of an SP or PA team and whether other students’ ideas are being heard (autonomy) and put into practice (freedom). These are some examples that illustrate this: A school manager expressed the staff’s focus on taking students seriously by involving them in decision-making processes. However, he acknowledged that they ‘tend to consult them after we as staff have detected the need and the ideas for the solution’ (i1). Another school manager mentioned working with student arenas: ‘where students can ask questions and make suggestions. The feasibility of their suggestions is discussed with them’ (i6). In several schools, healthy and active lifestyles are part of the culture ‘although that is not explicit in policy papers’ (i1). Another school explicitly stresses in its policy document that ‘reflections upon experiences are essential for learning’ (pd2). Another school’s policy takes this a step further when stating: ‘As a school, we have to create an environment where young people can learn democratic skills and about citizenship. A school environment with many rules without discussion or participation does not offer that space. It, therefore, starts with creating free space to distance oneself from daily occurrences to talk and reflect upon them and for trial and error’ (pd5). Teachers try to put this into practice, as illustrated by one teacher: ‘during my lessons, I try to adapt to what students want and need by working with smaller groups and different PAs’ (fg5). Also, teachers intend to ‘prepare PAs for students that cannot participate in regular PE lessons or PAs due to (temporary) impairments’ (i5). Particularly notable was the story of a PE teacher: ‘I was mentoring a student, a very sympathetic boy, who did not enjoy sports or PE lessons. The student was asked: “what is your active lifestyle”. He said: “I cycle to school each day, and I find that amazing. It is 8 km one way, and that is my PA moment. I enjoy that”. To me, that was an eye-opener’ (fg5). Experiences in other schools include working with a student council where ‘they discuss ideas’ (pd4) or the ‘set-up of a healthy school team in which students collaborate with teachers. The current team’s cohesion is excellent, inspiring to organise things together’ (fg2). In these teams and councils, students can develop their agency. However, some schools mention that they ‘do not actively ask or tune in on students’ needs or wishes’ (i3) or that it is ‘the SP and PA team who address students’ questions or wishes’ (fg1).

Concerning *family and community engagement*, all schools seem to reach out more or less to in-school and out-of-school collaboration with PA suppliers (autonomy). Particularly, in-school SP and PA team members play an essential role. In the Dutch context, the municipalities usually employ and post these SPs part-time in multiple social practices, ranging from schools to sports clubs and neighbourhoods. Their task is to link these social practices and translate sports and active lifestyles between these social practices. SPs work with a PA team in many schools, consisting of trainees in physical education and trainees from vocational education in sport and exercise. While SPs facilitate PA opportunities, they are not entirely free in their scope of action to follow up on students’ preferences (freedom) because they need to answer to the social practice that employs them. An illustrative quote was: ‘SP and PA teams actively ask students about their wishes’ (fg1) and ‘students are enthusiastic about the PA team’ (fg1). ‘The PA team is now almost institutionalised because they have a visible place in the school’ (fg2). Schools recognise the importance of the in-school SP: ‘the SP who acts like a ‘bridge’ between school and the sports club or different cultures would be most effective working with the older students. The biggest effort and focus should be on them because the motivation for PA lingers in that group, and students drop out of PAs. Maybe they will re-engage if you can provide a new perspective‘ (i4a). It is noteworthy that schools seem to make an external professional responsible for re-engaging students in PA and transferring PA to the out-of-school context. Schools recognise the impact of the home situation: ‘if children are not allowed to join a sports club, for whatever reason, it is hard to involve them in PA in school as well’ (i4a). Schools struggle to engage parents, often ‘limited to supporting transport to out-of-school PA events’ (i3).

To sum up, for these six schools in *physical education lessons, and before, during and after school*, teachers assume or experience that students are not (yet) capable of formulating their wishes concerning PA, and they try to find ways to let them experience new PA opportunities. Moreover, aspects of decision making, reflection and transfer are recognisable but not necessarily purposely addressed or interconnected. The schools’ *infrastructure* is an aspect that can allow for, or inhibit, PA-related agency. School policies can enable teachers to include agency in their pedagogical approaches. Still, it is often up to the staff member whether and how they operationalise this, including how they collaborate with the wider school community and PA suppliers.

### 3.2. Social Practice Dimensions per School

What stands out when capturing the dynamics within a particular school (see Table 2) is that most of the schools’ visions and pedagogical approaches, described in policy documents, hint at the development of students’ agency. However, the interviews and focus groups point out that operationalising this in practice is easier said than done, taking many shapes. Below, we sketch some striking observations of the particular dynamics for each of the six schools related to the three dimensions of social practice theory. Further details are reflected in Table 2.

In *School 1*, students may have complex home situations and negative experiences in different school systems. These situations often result in a lack of attention and financial means for PA at home (meaning). Within the school, student participation in PA is strongly encouraged by adults (based on a normative-pedagogical approach) (meaning). An SP introduced and supported a student PA team, putting them centre stage: they are visible in the school because of their nice sports outfits (materials), and their peers acknowledge their importance (meaning). Being part of the activity team implies a certain social status (meaning). However, the municipality is considering terminating the sponsorship of the SP (materials) for external reasons. Furthermore, organising PAs in and outside the curriculum can cause friction within the school, such as cancelling classes, replacing teachers, or noise disturbance (materials).

In *School 2*, active citizenship is part of the school’s core values, making a similar approach in PA internally consistent (meaning). PA possibilities at school are necessary to enthuse students to participate in PA (materials). Teachers strive to balance the free choice of PA with pedagogics on specific PAs because they are essential for developing (motor)skills or broadening horizons (meaning and competencies). There is proper coordination and collaboration between the SP and PE teachers (competences and materials). The school has a privileged physical environment within and around the grounds (materials). However, students’ freedom to use PA facilities is limited (materials). Students’ PAs depend on the choice options and the opportunities to put them to practice (meaning and competencies). The school creates student PA groups based on performance levels across educational levels. These groups support students to perform at their level and provide cohesion across academic levels (meaning). Students drop out of PA because they make different choices on spending their time, such as working part-time jobs (meaning). Parents participate in school PAs but do not feel and are not held responsible (meaning).

*School 3* aims to teach its students to become responsible and engaged citizens (meaning). Student participation is ingrained in the school’s modus operandi. Students are actively involved in many school processes and decisions, including PA. They are encouraged to take the initiative and work together to become who they want to be (competencies and meaning). They are invited to express themselves, ask questions and put ideas forward. This approach does not result in as many ideas as anticipated, probably because students lack the language or the ideas to ask for PA options that are unknown or not readily available (meaning and competencies). This school’s culture facilitates student participation in shaping PA options (competencies). School management is sports-minded, but teachers were not convinced of the need to focus on PA (meaning). The PA facilities are (and their use is) limited, but the school’s location is good and allows for active transport to school (materials). There is good coordination and collaboration between teachers, the SP and the PA team. The SP and PA team help students engage with sports and PA providers in the community (e.g., finances, contacts) (materials, competencies).

*School 4* is involved in a merger with another school. The school’s policy emphasises mastering talents and getting the best out of oneself (competencies and meaning). School management raises expectations about PA but does not follow through (meaning, materials and competencies). Limited PA infrastructure and dangerous traffic situations influence active transport (materials). Students are involved in the design of PA infrastructure (materials, competencies). PA initiatives are teacher-driven (competencies). According to the PE teachers, ‘students who need PA the most, lack the motivation’ (i4a) (meaning). Peer pressure seems to influence students’ participation in PA (competencies). The school offers structure and options to students (culture and rules), especially important for students with complex home contexts (meaning and materials). Students drop out of PA because they make different choices, especially when older (meaning). Teachers address the need for PA in parents–teacher conference evenings (meaning). The school has an SP and a PA team that structurally provide extra-curricular activities during breaks and before and after school. Activities are based on input from students but not elaborated in collaboration with them. Gathering the students’ input requires building a relationship of trust. SP also links students to PA providers outside the school; however, students’ interest is limited. There seems to be tension between the aims of PE teachers and SP regarding creating extra-curricular elective PA options and obligatory PE lessons. These activities depend on the efforts of the SP and PA team and are not structurally embedded in the school. The school works with sports associations to organise clinics. Students can skip other classes as a reward for PA (meaning).

*School 5′s* overall strategic policy documents emphasise a focus on talent development. PE teachers embraced this idea and made plans for PAs outside PE lessons. However, different management priorities prevented structurally embedding PAs outside PE lessons in school policies and practice (which is not necessarily the same as focusing on talent) (meaning materials). The efforts from teachers remain voluntary, which have disappeared under time pressure. Their PE teachers provide most PAs with little student input (competencies). Access to PA infrastructure is limited to PE lessons (materials). There is an active collaboration with other schools in the area. Still, other participants in the community around the school are hardly engaged in providing PAs (i.e., parents, sports associations) (materials, competencies). The school offered after-school PA options, but students choose differently (e.g., go home, gaming, work) (materials). The school has no budget for an SP and little time and space for developing PA-related agency competencies; students, especially those with no interest in PA, are not challenged to think about and act upon their PA-related objectives.

*School 6* strives for equal relationships between staff and students (competencies). Management is sport-minded and instrumental in improving PA facilities at the school (competencies and materials). The school’s vision on learning (combining challenges and offering space to experiment) matches an approach toward developing student agency (meaning). The school has no SP nor a PA team (materials). PE teachers increasingly employ their networks and creativity to create PA options outside PE lessons (competencies). The school has good working relationships with the municipality that facilitates the school prevention programme (competencies and materials). Organising PA outside the curriculum is challenging and causes friction within the school, such as cancelling PE lessons and depriving other students of their PA opportunities.

To sum up, concerning *meaning*, most schools’ policy documents refer more or less explicitly to their assumed role in educating students to become responsible, vital and engaged citizens who can achieve what they want. Concerning PA, an SP’s presence and *competencies* in working with PE teachers and students, seems an essential condition; the SP creates new, extra-curricular PA options, tunes into students’ objectives and wishes, and supports students with the transfer of PAs to other contexts. Regarding *materials*, the presence or absence of amenities and PAs in and around the school is crucial as the possibility/freedom to use them. The studied schools have not structurally embedded the reflection on the meaningfulness of these experiences for students and their transfer to other contexts in their practices. Although teachers challenge students to participate in PAs, this does not guarantee their participation due to different choices (e.g., part-time jobs, homework, or hanging around) or because PAs or facilities do not match their needs or perspectives.

## 4. Discussion

The results pertain to, on the one hand, the aspects of agency related to the elements of the WSA across schools and, on the other hand, schools as separate social practices. We will discuss these integrally. There is a wide diversity of school practices and how school managements create their balance of elements fostering students’ PA. The pattern of results indicates that the dynamics within each social practice differ between schools based on their particular vision, priorities, structure and setting. The question is whether these balances provide space to experiment with developing PA-related student agency. What do these schools do to facilitate the development of autonomy, giving choice options and freedom for students to choose in the pursuit of personal goals? The findings show that some schools have embedded the development of student agency in their integral social practice, whereas others have not. It could mean that all elements for developing PA-related student agency are present but not aligned with educating students for lifelong active lifestyles. This possibility is in line with Shove’s argumentation that the requisite elements for a particular social practice may co-exist but not be productively linked [31]. The missing links or elements in the social practice of the school may negatively impact the effectiveness and sustainability of the approach [32,38,39].

Although we did not include students as participants in the present study, we studied their perspectives elsewhere [12]. The findings from the current study are consistent with students’ perspectives: student perspectives on their PA are influenced by their motivations, what they deem meaningful and attractive and what they consider contextually possible and manageable. Inspiring teachers, beautiful playgrounds and innovative activities play important roles. Still, it was also evident that students have individual rationales for deciding whether or not to engage in PAs influenced by personal and contextual aspects, which are also confirmed by other studies, such as by Beni and colleagues [40].

We found a variety of illustrations of schools creating choice options and developing the autonomy component of agency. However, developing the freedom component seems less prominently addressed, although many requisite elements are present. In line with the findings from other studies [41,42], the three-elements model of social practice theory provided a helpful framework to study the configurations between elements to understand PA-related school practices.

Although they apply for a relatively standardised curricular programme, the studied schools form more or less distinctive practice realities based on the unique local configuration of materials, meaning and competencies. Thus, these schools deal with fostering student agency in different ways. From a social practice theory perspective, we propose an in-context analysis of student perspectives and reflection on their experiences, aiming at students navigating their participation in PA in different social practices, allowing for the transfer to future behaviour and employment of PAs in other life settings. Allowing students and their contexts to interact will most likely stimulate their (PA-related) agency, enabling them to develop their civic competencies and capability for lifelong PA. This contrasts with current public health and health promotion strategies that focus primarily on individuals making better choices, or changing contexts such that this leads to people making better choices.

Suppose autonomy and agency are considered valued outcomes of a school’s effort to enhance young people’s development into vital and responsible citizens. In that case, the school could benefit from integrating such outcomes explicitly in its policy, pedagogical approaches and infrastructural choices. This concurs with findings of a broader study into students’ general navigational agency in secondary education in Flanders, indicating that navigational agency is limited due to the lack of inner consistency of policy documents with no coherent pedagogical vision [43]. If PA choice options are available without specifically aiming for student agency or what they deem meaningful, students might still develop their agency. This notwithstanding, purposefully creating the conditions for students to co-create PA experiences, reflect on these experiences, and enable transfer to their contexts, increases the possibility for students to develop their critical consciousness and to discover PAs that fit their aspirations or purposes [1,9,29]. Schools integrating these three elements of co-creation, reflection and transfer may enhance the development of students’ PA-related agency contributing to the possibility of developing lifelong active lifestyles [44,45]. Studies into pedagogical approaches focusing on meaning-making and choice options indicate effectiveness at the student level and dependence on acceptance by teachers [46]. Several case studies show the feasibility and value of teachers adopting critical pedagogical approaches [45,46,47]. However, teachers’ effort to effectively adopt these approaches is considerable, should not be underestimated and is food for further research. It is influenced by the staff’s willingness, competencies and available resources in terms of support, vision, time, reflection on trial and error, strong leadership, and the pedagogical models used [47,48]. The pedagogical model for physical education related to health [49] could contribute to constructing a framework for this purpose.

### Strengths and Critical Reflections

A strength of this study is that it integrally unpacks school contexts by applying multiple mutually complementary theoretical lenses, notably social practice theory, WSA and agency theory. Thus, we gained nuanced insights into the socio-material configuration of people, structures, meanings and materials and their dynamics.

Despite our nuanced findings, some critical reflections are in order. In this study, we included only limited perspectives from the wider school community (i.e., parents, community PA providers, sports clubs). As students are part of multiple social practices, the inclusion and understanding of others into the wider school community could have enriched the insights and understanding regarding experiences bringing the ‘outside’ PA world into the school. Moreover, a certain congruence between these worlds is necessary to transfer PAs between the school and other contexts [50]. Therefore, for future research, we recommend also involving the wider school community.

Secondly, how schools address diversity in characteristics of students such as gender, ethnicity or socio-economic background was not accounted for in our study. Other research shows that school PA-related practices may include elements perceived differently by students with different personal characteristics, such as experiencing inequalities in obtaining recognition in sports and unequal opportunities, resources and support [43,51,52]. Therefore, it is reasonable to assume these characteristics influence students’ development of PA-related agency. Future studies could focus on how the culture, pedagogics, and language in school practices hamper or enable all students’ PA experiences and contribute to their PA-related agency.

## 5. Conclusions

To answer our research question, we conclude that the six schools have invested in developing students’ motor skills, opportunities to move in and around the school and introducing them to new PA experiences. This is in line with the contextual participatory aspects of agency by way of providing individuals’ choice options. However, to develop students’ capability for lifelong PA, it is also necessary that they are challenged to reflect upon their PA experiences and are supported in making the transfer to their own contexts, fostering their navigational agency. If not, students may be only ‘passive’ participants in school PAs without internalising what those experiences mean to them and how they can use and translate them to meet their own purposes in life. Our findings indicate that deliberation with students on the meaningfulness of these experiences and their transfer to other contexts is not structurally embedded in these school practices; when they do occur, they are generally but not necessarily picked up by the SP and the PA team who are temporarily assigned to a school.

## 6. Recommendations

For practice, we recommend that if schools embrace their responsibility to educate their students for lifelong PA, developing their agency should become part of their curricula and pedagogical approaches. This requires acknowledging students as active participants in various complex social systems that are interlinked, and of which the elements mutually influence each other. Moreover, teachers’ pedagogical approaches should foster students’ critical consciousness to act and reflect upon their experiences and contexts. This should include at least three elements: first, listening to students’ perspectives on their assets for PA and engaging them actively in shaping their PAs in the school context; second, facilitating students to reflect upon these PA experiences to discover what makes them meaningful and fit their aspirations or purposes; and third, supporting them in contextualising their experiences and transferring them to their out-of-school contexts, thereby transforming them. This approach requires a school culture where agency as a consistent pedagogical principle is embraced and practised.

The current study explored the links between the elements within a school practice at a particular stage in the process: after the school had partaken in a student participation project. For future research, we recommend further developing the potential of social practice theory as an interpretational frame for understanding how school PA practices evolve and develop over time.

## Figures and Tables

**Table 1:** Quotes attributed to the operationalisation of the whole-school components and the contextual dimensions of agency.

		Contextual Agency	
		School Practice Enhances Awareness of PA Options (Not Manipulated by Others; Autonomy and Context)	School Practice Provides Choice Options and Allows Students to Choose and Organise PA (Not Interfered with by Others; Freedom and Context)
**Whole-School (WSA) Components**	**Physical Education, PA during school time, PA before and after school**	− ‘We are a young school focused on what pupils need. We take them seriously and involve them in many things ranging from how to furnish the auditorium to PA.’ ‘Pupils are always involved although we tend to consult them after we as staff have detected the need and the ideas for the solution’ (i1). − ‘We challenge our students to use their talents to the best of their abilities’ (pd 1). − ‘A wide variety of PAs is announced through Instagram’ ‘they are in general designed by an adult, and students check the ideas. They do not have that many ideas themselves. You have to inspire and challenge them’ (fg2). − ‘School recently installed a PA team consisting of students and an SP’ (i2). − ‘We work with sports profile curricula. This means that children that enjoy PA are put together. At the same time, teachers can give specific attention to the groups with children with less interest in PA’ (i3). − ‘PE teachers used to organise PAs during recess, planning days for older or younger students and girls and boys. Two days in the week used to be unorganised’ (i4a). − ‘One of the things we offer is PAs on the school playgrounds during recess, organised twice a week together with the SP and the PA team’ (fg5). − ‘I do not know if we should give students only choice options. Sometimes they have to, I think. Maybe that is an old-fashioned thought, but sometimes you have to make students do things to experience things outside their comfort zone. I mean, they have to do something they are not good at or don’t like a priori, instead of them always choosing the same things’ (fg5). − ‘All first-grade students fill out a sports scan indicating how much they exercise, what they do and where. They discuss their results with their mentor. The school’s SP engages students in dialogue who are not yet involved in organised PAs outside the school, trying to support them with this’ (i5). − ‘The school has student arenas where students can ask questions and make suggestions. The feasibility of their suggestions is discussed with them’ (i6). − ‘Teachers were enthusiastic about the active involvement of students, asking them about their perspectives on their PA’ (i6).	− ‘We have talent hours as required, optional courses; students choose 4 out of 12. It is fairly supply driven’ (i1). − ‘Our final course students teach PE in groups of 3 students at the local primary school. PE teachers design the lesson, and students look up ways to operationalise the aims of the lesson’ (i3). − ‘The third graders are involved in organising school tournaments’ (i3). − ‘During schooltime, students like to participate in PAs; it is more difficult outside school’ (i5). − ‘As a school, we participate in many local and national tournaments, and we create student teams across school levels which contribute to building citizenship’(i5). − ‘We do not have a student council focused on PA yet, but it would be great to create one’ (i5). − ‘In 4th grade, they independently teach each other during PE lessons’ (fg5).
	**School’s infrastructural (im)possibilities**	− ‘We do not own the sports amenities, so we always have to clear out for other users’ (i1). − ‘The obstacle run and laser game entailed using the whole school building differently. This caused some friction with colleagues because of some noise disturbance. It has been organised several times’ (i2). − ‘The school is located in a rural community. Children come to school by bike. The school provides bike sheds and technical support’ (i3). − ‘Students were invited to provide input for the new school playground. We organised a competition and invited the three winners to co-design with the school director and architect’ (i4). − ‘An e-sport room was created with static bicycles and a dance floor attracting die-hard gamers. You have to keep taking different initiatives to trigger different groups of students.’ (i4a). − ‘We have new amenities and fantastic sports halls for gymnastics a boulder wall, and we are designing an active playground’ (i5). − ‘Management was crucial in accomplishing a sports court in the school playground’ (i6)	
	**Staff**	− ‘The SP confers with the PE teachers’ (i1). − ‘The school culture stimulates an active and healthy lifestyle although not explicit in policy papers’ (i1). − ‘Every student is curious. Learning implies that the student can see connections and recognise the meaning. This requires a safe and inspiring school environment. Reflections upon experiences are essential for learning’ (pd 2). − ‘If students are invited to co-think, they are acknowledged and become important in the eyes of other students’ (fg2). − ‘Every child has the right to discover and develop its talents’ (pd 3). − ‘The workload is hefty. This makes it hard to develop ideas into plans and practice. It is all voluntary extra work. It sounds ‘soft’, but you need to feel facilitated and supported by the management’ (fg3). − ‘We don’t actively ask or tune in on students’ needs or wishes’ (i3). − ‘Students’ attitudes are evaluated and weighed equally with their performance’ (i4a). − ‘Motor skills of students vary a lot, especially when they start their school career’ (i4a). − ‘During my lessons, I try to adapt to what students want and need by working with smaller groups and different PAs ’ (fg5). − ‘We are preparing PAs for students that cannot participate in regular PE lessons or PAs due to (temporary) impairments’ (i5). − ‘As a school, we have to create an environment where young people can learn democratic skills and learn about citizenship. A school environment with many rules without discussion or participation does not offer that space. It, therefore, starts with creating free space to distance oneself from daily occurrences to talk and reflect upon them and for trial and error’ (pd5). − ‘You need people with a drive to put in the extra effort. Then you create enthusiasm amongst colleagues and students. Now you have a great team, but that has not always been the case’ (i5). − ‘Our management is sport-minded. That helps’ (i5). − ‘If all the PAs on offer are optional and students can choose freely, they will not choose the subjects they are not interested in or feel motivated for. On the one hand, students should be able to develop according to their personality freely, but on the other hand, you get a kind of polarisation in the fundamental education all students should receive concerning motor skills and fitness or for example lifting techniques’ (i4).	− ‘The SP and PA team address students’ questions or wishes’ (fg1). − ‘Each year we set up a healthy school team where students collaborate with 3 teachers, including a PE teacher. The cohesion in the current team is excellent, which inspires to organise things together’ (fg2). − ‘Children choose this school because of the sport focus in the curriculum’ (i3). − ‘Ideas are discussed in the student council’ (pd4). − ‘Students want to belong to a group, and they follow whatever the peer leader does or says. To engage a group in something, you need to detect the peer leaders and enthuse them to reach the other group members’ (i4a). − ‘Once they are in their final year, then they do not do anything anymore (related to PA *red*) Their final exams, part-time jobs, scooters, smoking and boy- or girlfriends. That is their only focus’ (i4a). − ‘Extra PAs and tournaments are organised and supervised by PE teachers. This sometimes creates problems for other lessons and students missing out on their PE lessons’(i6).
	**Family and community engagement**	− ‘Engagement of parents and students makes our schools stronger. We actively involve them in our policymaking through councils and social media and thematic meetings’ (pd1) − ‘SP and PA teams actively ask students about their wishes’ (fg1). − ‘Students are enthusiastic about the PA team’ (fg1). − ‘The home situation has a big impact; if children are not allowed to join a sports club, for whatever reason, it is hard to involve them in PA in school as well’ (i4a) − ‘Parents are not actively involved in organising school PAs. They merely help out by driving students to tournaments or games’ (i3).	− ‘The PA team is now almost institutionalised because they have a visible place in the school’ (fg2). − ‘The SP who acts like a ‘bridge’ between school and the sports club or different cultures would be most effective working with the older students. At least, that is where the most extensive effort should be focused on. Because it is in that group, motivation for PA lingers, and students drop out of PAs. If you can provide a new perspective, maybe they will re-engage (i4a) − ‘.. also with the out-of-school tournaments. Other schools assemble students teams in no time, and I have to put in much effort to persuade students to participate’ (i4b) − ‘I was mentoring a student, a very sympathetic boy, who did not enjoy sports or PE lessons. He was asked, “what is your active lifestyle” and he said: “I cycle to school each day, and I find that amazing. It is 8 km one way, and that is my PA moment. I enjoy that”. To me, that was an eye-opener. I thought: “Wow, this is how it can be’ (fg5).
	**Community infrastructure**	− ‘The roads surrounding the schools have been adapted to make them safer with speed bumps’ (i5). − ‘The school is located in a rural area, and students come to school by bike despite dangerous traffic situations. We are negotiating with the municipality to make the roads surrounding the schools safer’ (i6).	− ‘School 1 is located on the edge of the city. Many children come to school by bike’ (i1). − ‘In School 2 students have adopted a roundabout or traffic situation and they prepare plans for improvement and safety and present them to the municipality’ (i2). − ‘It is a rural school, and students arrive by bike from the surrounding villages. Roads and bicycle lanes are separated’ (i3).

Legend: s1 = school 1, etc.; i1 = interview 1, etc.; fg1 = focus group 1, etc. SP = sport professional; PA team = physical activity team; PE = physical education.

**Table 2:** School characteristics as social practice.

	School 1	School 2	School 3	School 4	School 5	School 6
**Meaning**	A Roman Catholic school. Students are prepared for an independent life in society through individual and collaborative learning. Students learn to take responsibility for their learning process, society and nature. School policy fosters human dignity, well-being, solidarity, justice and subsidiarity. The schools’ pedagogical and didactical *Leitmotiv* is that for students to develop into mature and well-balanced adults, they need to acquire knowledge and develop their socio-emotional, creative, spiritual, physical and technological competencies.	The school’s vision on the importance of pursuing citizenship competencies is deeply embedded in school practice. Core values: 1. Trust in students’ and teachers’ development; 2. Aiming at increasing responsibility; 3. Accepting differences between people; 4. Providing freedom in mutuality. The school aims for each student to develop an independent identity free from the judgements of others. They form their judgements, are critical and think for themselves. The school is a place to meet and connect.	The schools embrace the responsibility to teach their students competencies to become responsible and engaged citizens. One of the critical elements of the schools’ policy is that students fully use their talents. Students are taken seriously; staff seek their opinion and ideas and encourages them to ask questions.	Mission: every student masters his/her talents. It offers students the opportunity to become active citizens and to excel. All students learn that employing their talents means participating fully in society. Core values are: - Passion and engagement; - Responsibility and reliability; - Civilised behaviour and respect for each other, nature, religious beliefs and worldviews, diversity in cultural backgrounds and sexual orientation; - Professionalism.	Vision: focus on offering opportunities to students; each student has the right to discover and develop his or her talents. Students are facilitated in making informed study choices. Core values are: ambition, openness, safety, a focus on collaboration, pride.	Mission: every student can fully develop into a self-conscious, autonomous, socially involved young person with entrepreneurial qualities who contributes to society sustainably, now and in the future. Motto: space for talent. Core values and vision on learning: - We believe in individuals’ capacity to develop - everybody is able and should take responsibility. - Learning is about combining challenges and offering space.
**Materials**	In- and outdoor sports facilities and playground, only available under the supervision of an adult (teacher, parent). The bicycle shed is out of sight (dangerous, prone to vandalism, etc.). Sometimes the whole school building is used as a playground for specific PAs. Most students use active transport (bicycle) to come to school.	Excellent PA amenities, in- and outdoors, allowing active transport to school (cycling). Improved bicycle shed and made the traffic situation safer in front of the school. However, amenities are not always available for students to use, i.e., breaks, or before or after school, because of other PE classes, neighbours, supervision requirements. Most children cycle to school; others have a seasonal bus pass or are brought to school by car (in winter).	The school uses in- and outdoor facilities, most of which are owned by the municipality (school is not in control of their use). There are plans for new construction including sports facilities. Most students cycle to school.	Lack of PA infrastructure. Playground used for PAs during breaks and sometimes for PE lessons which can be seen from the classrooms: using it for PA/PE causes embarrassment. An E-fit classroom is available for individual (gaming) exercise. Students can use the infrastructure under supervision. Students cycle to school—dangerous traffic situation near the school; supervised in the afternoon.	In- and outdoor sports facilities allow children to cycle to school. School amenities are not available for PA outside PE lessons because they are in (preparatory) use for other PE lessons or by sports associations (after school). Most students cycle to school.	In- and outdoor sports facilities, including a multifunctional sports court. Students can use them, both under supervision and freely. The court is inside school gates, so unavailable outside school hours (prevent damage by other users). Natural school surroundings are used. Most students cycle to school.
**Competencies**	- Management in favour of making students co-owner of the school. Reflection on experiences and discussing vital questions is fundamental. Teachers and management inspire, support, listen and lead by example. Awareness of doing a lot for students but not with them. - Students can choose from elective study profiles and a wide variety of Pas; - A healthy school working group in which students participate; - The school’s activity team (AT) was set up: a group of students in charge of co-organising PAs based on input from other students and supervised by a PE teacher; - AT works together with an SP who co-facilitates PAs; - A driving force is one of the PE teachers who organises a wide variety of PAs and uses social media to inspire them to participate; - Collaboration with other PA providers is challenging because of their voluntary structure; - Traffic education includes students adopting a nearby roundabout; - Teachers’ perspective is that students find it hard to think of and express what they want regarding PA; - Students’ initiatives are welcome but pose supervision problems, sometimes tackled with competent parents; - Management encourages teachers and students to push their boundaries	- Management is sport-minded; - Students have different (extra) in- and extra-curricular PA choice options; older students can choose and teach PAs; - Students’ perspectives on their PA can be eye-openers for teachers; - PE teachers are enthusiastic; they lead by example, provide choice options in and outside PE lessons, involve students in PA in and outside their comfort zone. Students can choose but are also engaged in PA that they do not necessarily like at first glance. Students are marked on progress rather than performance; - Student groups are formed based on their performance level; - PE teachers’ intend to provide PA options for students with special needs; - An SP and PA team provide extra-curricular PA options in breaks, and before and after school, based on a student scan and input. The SP also links students to PA providers outside school.	- Management is sport-minded. Teachers resist PA project initiatives, the need for which was not well explained by the management; - School provides choice options and electives for students. The management perspective is that students do not always say what they think, partly because they are content with what is available, partly because they lack the examples or the language. Students are challenged to take the initiative and work together to become who they want to be; - An SP and PA team provide extra-curricular PA options in collaboration with PA providers in the community. The school’s students inform these PAs. The SP also helps students with their PA wishes and needs in different ways (looking for finance, involving their local network to create PA options, etc.).	- Management is open to but has no specific interest in PA; they do not always live up to raised expectations; - A variety of PAs are offered by either PE teachers or SP and the PA team. They provide extra-curricular activities during breaks, and before and after school, informed by students through student council and questionnaires (but not developed with them). These activities depend on the efforts of the SP and PA team, and are not structurally embedded in the school. They also work with sports associations’ organising clinics. SP links students to PA providers outside school; students’ interest is limited; - Some classes or PAs are elective (students can develop their own choice of talents); others are mandatory as part of the core curriculum; - Students contributed to the layout and design of a multifunctional sports court and school playground; - Grading is based on attitude, motivation, and performance; - Students often have complex home situations; - School is committed to engaging parents, but their role is limited.	- School offers several pre-established choice options and mandatory extra-curricular PAs; - No PAs are offered in breaks or before or after school; - Students are organised in groups according to their competencies to be challenged at their level; - Management favours PA but does not prioritise it in managerial choices. Focusing on PAs outside PE lessons is a voluntary effort by the teachers, not facilitated by management; - Students develop and carry out their lessons and tournaments for primary school classes and younger schoolmates as part of their curriculum; - Students are not involved in the development of other PAs or the playground’s design; - After school PA options are offered, but students choose differently (go home, gaming, working, etc.) - Teachers inspire by example. They offer sports clinics and other PAs to inspire their students.	- Management is sport-minded and instrumental in establishing the multifunctional sports court. - The school works with a student arena where students can ask questions and build self-confidence to put things they consider important on the agenda. - Teachers are growing into providing inspiring alternative PA activities collaborating with nearby PA providers. They do not involve students in the choice options or the organisation. - The school has a good working relationship with the municipality in organising ‘prevention evenings’ in which all kinds of health topics are addressed. Parents are actively involved in the process of learning healthy and active lifestyles.

Legend: SP = sport professional, PA team = physical activity team, PE = physical education.

## Data Availability

The data presented in this study are available on request from the corresponding author. The data are not publicly available due to the nature of the study, which required establishing links between interviewees, their schools and policy documents.

## Note

Gwendolijn M. M. Boonekamp undertook this study as part of a Ph.D. in Health Sciences under the supervision of Professor Carlos Alvarez-Dardet Diaz at the University of Alicante and as a member of the Research Group for Social Support and Community Care at HAN University of Applied Sciences.
